# Reliable 3D video streaming considering region of interest

**DOI:** 10.1186/s13640-018-0273-y

**Published:** 2018-06-07

**Authors:** Ehsan Rahimi, Chris Joslin

**Affiliations:** 1 Department of Systems and Computer Engineering, Carleton University, 1125 Colonel By Dr, Ottawa, K1S 5B6 ON Canada; 2 School of Information Technology, Carleton University, 1125 Colonel By Dr, Ottawa, K1S 5B6 ON Canada

**Keywords:** 3D stereoscopic videos, Color video, Depth map image, Reliable multimedia streaming, Multiple description coding, Object identification

## Abstract

3D video applications are growing more common as communication technology becomes more predominant nowadays. With such increasing demand for the 3D multimedia services in either the wired or wireless networks, robust methods of video streaming will be introduced to show more favorable efficiency outcomes since packet failure is an integral characteristic of communication networks.

This paper aims to introduce a new reliable method of stereoscopic video streaming based on multiple description coding (MDC) strategy. The proposed multiple description coding generates four 3D video descriptions considering the interesting objects contained in the scene. To be able to find the interesting objects in the scene, we use two metrics from the second-order statistics of the depth map image in a block-wise manner. Having detected the objects, the proposed multiple description coding algorithm generates the descriptions for the color video using a nonidentical decimation method with respect to the identified objects. To show how much reliable the proposed MDC method is, this article assumes that due to the unreliable communication channel, only one description, among four encoded descriptions, is delivered to the receiver successfully. Therefore, the receiver needs to estimate the missed descriptions’ data from the available description. Since the human eye is more sensitive to objects than it is to pixels, the proposed method provides a better visual performance in view of its subjective assessment. Although, the objective test results verify the fact that the proposed method provides an improved performance than the Polyphase SubSampling (PSS) multiple description coding and our previous work using pixel variation. Regarding the depth map image, the proposed method generates the multiple descriptions according to the pixel prediction difficulty level. The considerable improvement achieved by the proposed method is shown with the peak signal-to-noise ratio (PSNR) and Structural SIMilarity (SSIM) simulation result.

## Introduction

Multimedia communications such as broadcast TV, TV on demand, video conference, and live stream event services have grown exponentially over the last few years making it difficult for operators to keep up with the increasing demand for bandwidth capacity for streamlining videos; although, with the advent of High-Efficiency Video Coding (HEVC) and the later video streaming algorithms such as parallel framework on many-core processors presented in [1, 2], the major pace to deliver high-definition (HD) video to consumers has been done effectively. According to CBC news in 2015, downloading of video content from the North American Netflix has doubled in 5 years from 35% in 2010 to 70% in 2015 [3]. This elevated data traffic has been caused by the ubiquitous accessibility of multimedia applications. To add even more pressure to bandwidth capacity, 3D/multiview videos are now becoming more popular among multimedia users. Consequently, more bandwidth is required with the more popular 3D/multiview videos with its depth information or multi views.

Smolic and Kimata defined 3D videos as “geometrically calibrated and temporally synchronized video data” [4], which means that more memory and bandwidth are required to store or stream 3D/multiview videos, respectively. Even though the technology for producing memory has been developed in the last decade, it is still a challenge to save the enormous volume of 3D/multiview video data effectively. More importantly than saving video data are the stream immersive video restrictions of insufficient bandwidth and unreliable communication. Besides, image content analysis which is a promising research area for the intelligent applications is more difficult for 3D/multiview videos; although, different techniques such as hashing algorithm have been proposed to improve the efficiency and complexity of image content analysis [5, 6].

One major problem with the stream of a video is errors that happen due to the unreliable communication in both wired or wireless networks [7–14]. In the wired networks, errors can occur due to packet loss, corruption, congestion, and large packet delay, whereas in the wireless networks, unreliable communication can stem from temperature noise and interference that exist in the physical environment. When dealing with immersive videos, the increase of the data traffic load will consequently produce data congestion. Therefore, the serious packet failure problem needs to be addressed since such errors on the delivered video diminish the viewing quality experience. To avoid such errors, an error-resilient method of data transmission is used by the encoder.

Generally, there are usually three methods in the communication systems to avoid packet failure: Automatic Repeat reQuest (ARQ), Forward Error Correction (FEC), and Error-Resilient Coding (ERC) [7]. The first method, the ARQ approach, requires a network with feedback capability, and as a result, it is not suited for real-time or broadcast applications. The second method, the FEC approach, is designed to cope with a specific amount of noise error making it impractical for noise variances that exceed the threshold level. The third method, the ERC approach, is the approach of choice in this paper because of its resiliency against packet corruption or noise feature. This resiliency is achieved through redundancy bits added to the data stream. There are a number of methods where redundancy can be introduced to the stream including Reversible Variable Length Coding (RVLC), intra refreshment, Flexible Macroblock Ordering (FMO), layered coding (LC), and multiple description coding (MDC). Among these methods, RVLC used in H.263 lacks coding efficiency [15]. Intra refreshment and FMO (used in H.264) are beneficial for the channels with low noise variance. With layered coding, the layers are not separately decodable resulting in performance dependency upon lower layers to be without error. Therefore, the layered coding is less advantageous for error-prone environments. The multiple description coding method is our method of choice due to its suitability for large noise power channels. MDC avoids packet failure because it creates multiple complementary and separately decodable descriptions.

With the MDC method, video data is partitioned into several descriptions and then encoded separately. The descriptions are then streamed through the network toward receiver(s). In the receiver, there are two different types of decoder—the side decoder and central decoder. The receiver chooses one of the two decoders based on the availability of error-free descriptions remaining.

The instance that all of the descriptions are received successfully is when the central decoder is activated. Otherwise, the side decoder will be activated when only a few error-free descriptions are received. The MDC method is best recognized for its error robust property at the expense of compression ratio as it adds redundancies in its temporal, spatial, or frequency domain. With the temporal MDC method, usually, two descriptions are produced in order to avoid a drop in the coding efficiency. The drop in the coding efficiency is reflected when more than two descriptions are used because the distance between the assigned frames to each description is increasing resulting in the motion prediction being less effective [8, 16]. When the network is very noisy, a higher number of descriptions are required. Therefore, the temporal MDC method is no longer a suitable technique. The frequency MDC method partitions Discrete Cosine Transform (DCT) coefficients between video descriptions. Because DCT transformation provides independent components, the descriptions will be less dependent. To maintain the correlation of the descriptions, an extra transformation like Lapped Orthogonal Transformation (LOT) needs to be applied. Therefore, the complexity of frequency MDC methods is higher than that of both the spatial and temporal MDC methods, respectively. With the spatial MDC method, each video frame is partitioned into several lower resolution sub-images using Polyphase SubSampling (PSS) algorithm [7, 17, 18]. It is worth mentioning that with a simple spatial MDC method, there is no precise adjustment tool over the redundancy in order to control the side quality [7, 17, 18]. This means that there is no control for the redundancy increase resulting in higher resistivity to compensate for the higher noise level.

To improve the basic spatial MDC methods, Tillo and Olmo introduced a new MDC algorithm called “least predictable vector directional multiple descriptions coding” [10]. This approach basically copies the least predictable parts of the frame to all descriptions. Their simulation result shows that this method improves the side quality when compared to previous PSS-MDC method although the new method provides more redundancy. Tillo and Olmo obtained better quality for higher noise level at the expense of less coding efficiency and greater algorithmic complexity.

Shirani also presented a non-linear PSS-MDC method which investigated its performance by evaluating the case where there were one or more missing descriptions [19]. According to his work, some parts of a frame which are more important called region of interest (ROI) were sampled with a greater rate (based on an exponential equation) compared to other parts of the frame. In other words, descriptions include more information regarding the ROI parts of the frame resulting in an enhancement of the side quality. More importantly, this method provides for greater performance with regards to the subjective assessment by the human eye since objects and not pixels are more emphasized. Although Shirani’s method provides for the enhancement of the side quality, he did not discuss how the ROI parts of a frame were detected which is important when involving fast video contents or live video streaming. In this paper, we provide a new spatial MDC algorithm that adds redundancy to the descriptions more practically for 3D videos.

To apply the MDC method for 3D videos, the depth map image also needs to be partitioned into different descriptions. It is worth mentioning that the depth map image mainly contains depth information of the scene’s objects. Because of the nature of the real objects, depth information of 3D scenes rarely contains high-frequency content. Consequently, the depth map image can be effectively compressed effectively resulting in saved bandwidth and disk space [20, 21]. To improve compression, Karim et al. have shown that the downsampled version of the depth map image provides an adequate reconstruction of the 3D video in the receiver [22]. They have experimented with the spatial MDC method for 3D videos using color plus depth map image representation. Karim et al. have carried out experimental tests with a scalable multiple description coding approach arriving at the same result. Therefore, it can be said that downsampling of the depth map image does not cause a considerable degradation in the quality of a reconstructed video. This is due to the fact that the depth map image includes low-frequency contents or, more precisely, the depth values of adjacent pixels are similar. Consequently, one can state that the neglected pixels during downsampling can be better predicted. Liu et al. utilized the fact of having similar depth values of pixels for real objects and introduced a texture block partitioning algorithm in order to perform their MDC algorithm for wireless multi-path streaming [8].

## Proposed method

**Fig. 1:**
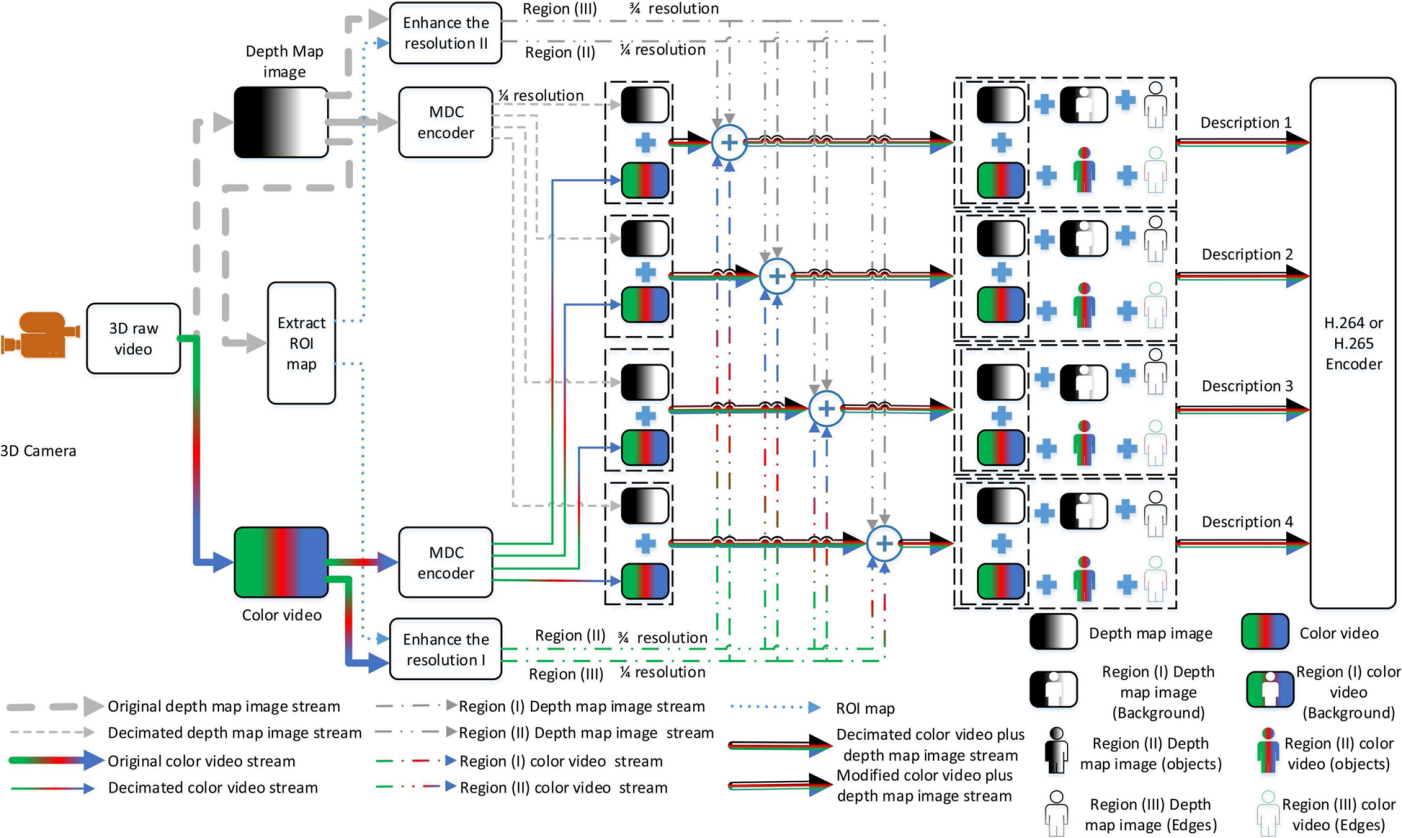
Block diagram of the proposed method. This figure shows how the proposed encoder works. It includes three parts: Part (I) This part of the encoder is responsible to extract the map of different regions of interest and feeds part III of the encoder (more detail in Section 2.2). Part (II) This part separates the depth map frame and the color video frame. Then, every frame of both the depth map and color video frame are decimated in the pixel domain to created four descriptions. Each description contains one pixel of every block of size 2 × 2 (see Section 2.3). Part (III) This part enhances the resolution of different regions of interest as explained in Section 2.4

This section describes the new proposed multiple description coding applicable for 3D videos. Before starting to describe the proposed algorithm, we need to define a metric to identify ROI and clarify how it can be used for the purpose of ROI extraction. In this paper, we use two metrics as explained in Section 2.1. An overview of the proposed encoder is shown in Fig. 1. As can be seen in this figure, the new proposed MDC algorithm consists of three steps: the extraction of the map for ROI, the Polyphase SubSampling MDC, and the enhancement of the descriptions. Firstly, the extraction of the map for ROI splits each 3D raw frame into a 2D color frame and a grayscale depth map frame, then looking for different regions of the frame using the depth map image. The process of extracting the ROI is described in Section 2.2. Secondly, the Polyphase SubSampling MDC as explained in Section 2.3 creates four sub-images using Polyphase SubSampling (PSS) from both the color and the depth frame separately. Thirdly, the enhancement of the descriptions is achieved through the combination of different regions of the frame with different resolutions obtained from the color and depth map streams. This step of the new spatial MDC algorithm is fully described in Section 2.4.

### ROI extraction metrics

In order to be able to recognize which part of the frame is more important or ROI map extraction, a metric needs to be defined. To this end, we calculated the average of the absolute variations for pixels’ values found in the depth map image in a blockwise manner:

1 $$ PV_{i} = \frac{1}{N_{i}}\sum\limits_{j=1}^{N_{i}}|D_{j}-\mu_{i}|  $$

where *μ*_*i*_ is the average of depth values for block *i*, i.e.,

2 $$ \mu_{i} = \frac{1}{N_{i}}\sum\limits_{j=1}^{N_{i}}D_{j},  $$

and *PV*_*i*_ stands for the pixel variation of block *i*; *D*_*j*_ is the depth value of pixel *j* in the *i*th block and *N*_*i*_ is the total number of pixels in block *i* (i.e., *j* = 1,2,...,*N*_*i*_). Hierarchical block division algorithm is further explained in Section 2.2.

Generally, *PV* of a block is a positive value that can be changed from zero to infinity. Blocks with large *PV* are probably related to several objects or edges. Because the depth information of an object contains low-frequency contents naturally, the depth values of pixels for an object are similar. Blocks with very small *PV* is related to the far-distanced background or the planar objects, for example, a wall.

**Fig. 2:**
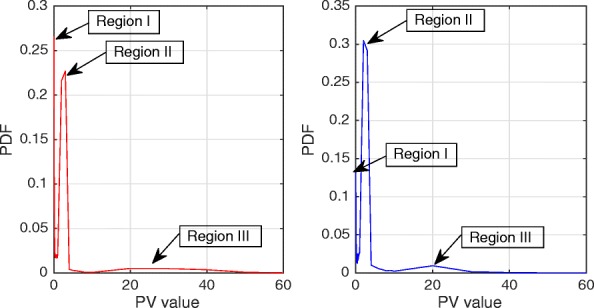
PDF of the depth’s pixel variations. The probability density function of the depth’s pixel variations for the first frame of video “Interview” (left) and “Orbi” (right). As can be seen, three regions are distinguished for both video tests. They are blocks with depth variation very close to zero, between zero and one, and greater than one (approximately)

**Fig. 3:**
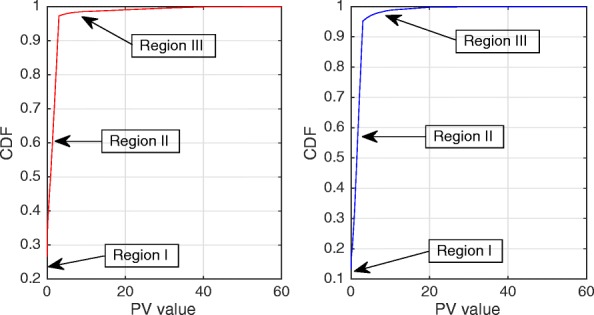
CDF of the depth’s pixel variations. The cumulative density function of the depth’s pixel variations for the first frame of video “Interview” (left) and “Orbi” (right). Like its PDF, three different regions can be recognized for both video tests

**Fig. 4:**
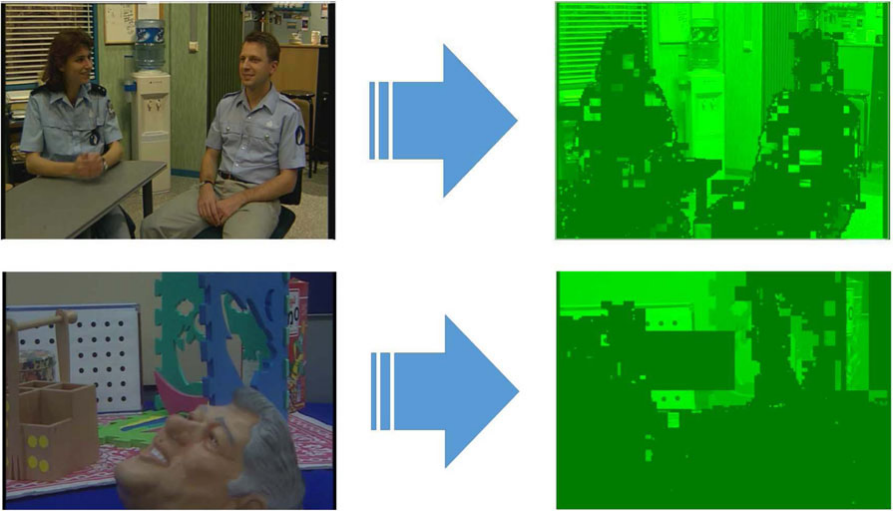
Region I. The identified region I using metric *PV* for two sample video tests

**Fig. 5:**
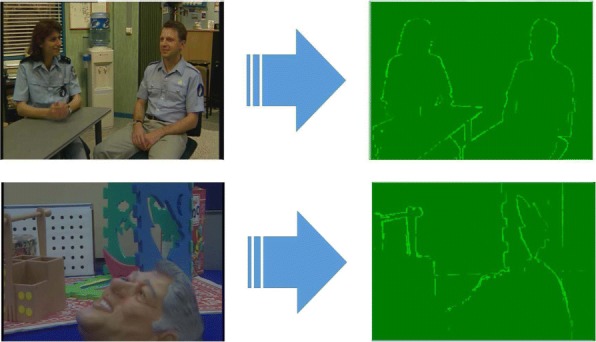
Region III. The identified region III using metric *PV* for two sample video tests

**Fig. 6:**
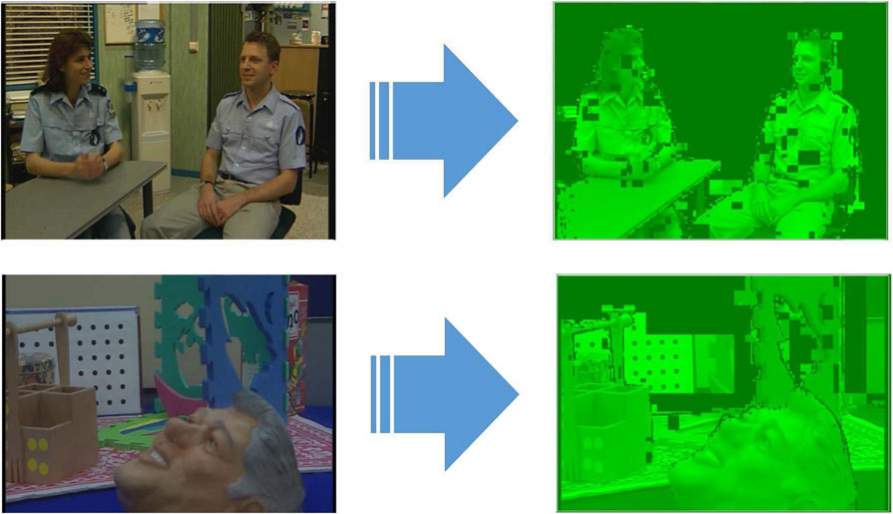
Region II. The identified region II using metric *PV* for two sample video tests

Figures 2 and 3 show the Probability Density Function (PDF) and the Cumulative Density Function (CDF) of *PV* for two sample videos entitled “Interview” and “Orbi.” In Fig. 2, the PDF (*Pr*(*PV* = *x*),0 < *x* < +*∞*) of the *PV* values for these two videos depict pixels that are classified into the following three regions: regions I, II, and III. Region I demonstrates that pixels of the frames have very low depth variation. Due to this very low variation region, the CDF is shown to start from a nonzero point in Fig. 3. Figure 4 shows that the region I pixels belong to the first frame of both videos, Interview and Orbi. Region III pixels have a large depth variation and are most likely depicting the edges of different objects. Region III pixels in Fig. 2 are showing a small peak when the *PV* value is greater than 5. In Fig. 3, region III is started from the point that the slope of the CDF graph changes from steep to moderate. In Fig. 5, region III pixels in the first frame of both video Interview and Orbi are being shown. The remaining part of the frame is region II. Region II in Fig. 6 is showing the objects of interest depicted from the video Interview and Orbi, respectively. Region II is relative to the PDF found in Fig. 2 for its second peak and CDF in Fig. 3 for its steep slope. For the remainder of this paper, regions I, II, and III will be named background region, region of interest (or interesting objects’ region), and edges’ region, respectively.

As can be seen in Figs. 4, 5, and 6, the performance of the ROI extraction algorithm is not high enough. For example in Fig. 6, there are some missed blocks in the middle of interesting objects, or some blocks have been detected as ROI which are in fact non-interesting objects in the background. This is due to the fact that it is not appropriate to compare the metric introduced in Eq. 1 (*PV*) for different blocks. In other words, pixel variations of respective blocks found in different scales need to be normalized. Therefore, we define a new metric (*CV*) as the ratio of pixel variation (*PV*) to the mean *μ*, also known as coefficient of variation (CoV):

3 $$ CV_{i} = \frac{PV_{i} }{\mu_{i}},  $$

where *CV*_*i*_ is CoV for the block *i* within a depth map image. *PV*_*i*_ and *μ*_*i*_ have already been defined in Eqs. 1 and 2, respectively.

Similar to *PV*, the *CV* has a positive range of zero to infinity. When *CV* of a block equals one, then the depth values of that block have the same mean and standard deviation values. It can also be argued that blocks with large *CV* values are probably related to several objects or edges while blocks with very small *CV* values are related to the background of the video frame. Consequently, they are not the interesting part of the frame that the ROI extraction algorithm is looking for.

**Fig. 7:**
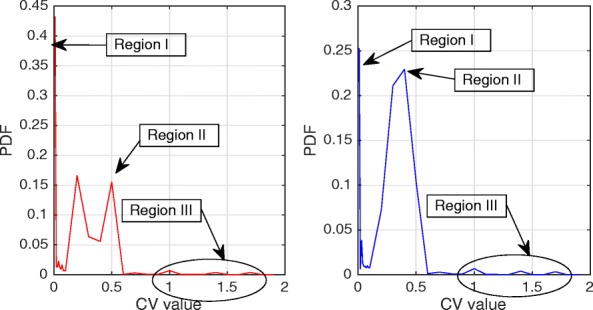
*PDF* of the depth’s coefficient variations. The probability density function of the depth’s coefficient variations for the first frame of video “Interview” (left) and “Orbi” (right). The regions are more distinguishable compared to the *PDF* of the depth’s pixel variations (especially region II)

**Fig. 8:**
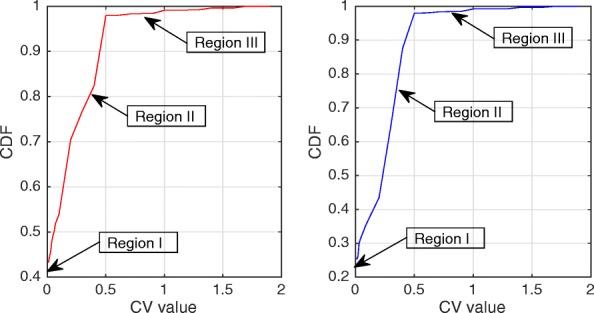
*CDF* of the depth’s coefficient variations. The cumulative density function of the depth’s coefficient variations for the first frame of video “Interview” (left) and “Orbi” (right)

**Fig. 9:**
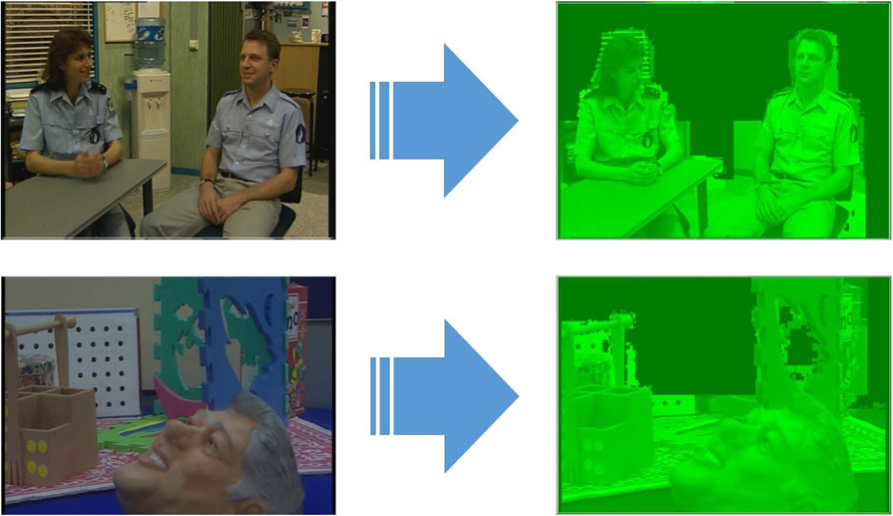
Region II. The identified region II using metric *CV* for two sample video tests. Compared to the region II identified with the help of metric *PV* and shown in Fig. 6, it is more accurate

The typical Probability Density Function (*PDF*) and Cumulative Density Function (*CDF*) of *CV* values for video Interview and Orbi are shown in Figs. 8 and 7, respectively. Like Figs. 3 and 2, the same argument is applicable for *PDF* and *CDF* as shown in Figs. 8 and 7. A sample of detecting the map for region II (interesting objects) using the CV metric is shown in Fig. 9. As can be seen in this figure, the identified ROI with *CV* values is considerably more accurate than the similar region shown in Fig. 6.

### ROI extraction algorithm

As shown in Fig. 1, the first step of the proposed encoder is to determine which part of the frame is more important. One important issue in this process is its requirement for a low-complexity algorithm in order to realize the interesting objects in the frame. Since color video frames contain a large variety of frequency components, it is more convenient to extract the ROI map using the depth map image. Generally, the depth map image includes two major contents:

- First, low-frequency contents; since the depth information of natural objects is usually similar, it can be said that the depth map image predominantly contains low-frequency contents.
- Second, edges; there are usually sharp edges in the depth map image due to the different depth information of foreground and background objects.

The ROI extraction algorithm uses the characteristics of the depth map image and extracts the map of ROI using one of the metrics explained in the previous section.

**Fig. 10:**
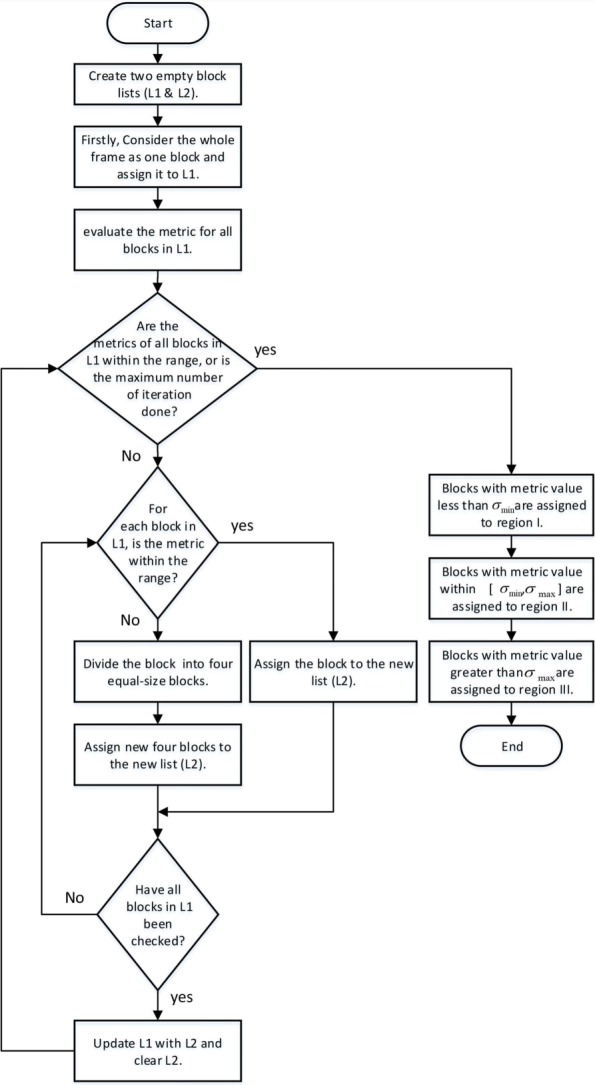
Proposed ROI extraction algorithm. The algorithm divides all the blocks that their metric values are not in the ROI range (between *σ*_*min*_ and *σ*_*max*_). The process will continue until there are not any blocks of size greater than 2 × 1 or 1 × 2 and having the metric value out of the ROI range

Figure 10 shows the algorithm that identifies the objects proposed by this paper. In this figure, the ROI range is defined as the distance between *σ*_*min*_ and *σ*_*max*_. *σ*_*min*_ is the threshold which is used to separate the very far objects in the background from the interesting objects, and *σ*_*max*_ is the limit used to detect edges of the interesting objects. Clearly, the ROI range is different for two metrics that were introduced in Section 2.1 and shown by $\left [\sigma _{min}^{PV},\sigma _{max}^{PV}\right ]$ and $\left [\sigma _{min}^{CV},\sigma _{max}^{CV}\right ]$. The minimum thresholds are set so that the very far background can be separated from the interesting objects. Based on our experiment results in Figs. 2, 3, 7, and 8, $\sigma _{min}^{PV}$ and $\sigma _{min}^{CV}$ can be a value approximately between [0.1 0.3] and [0.01 0.1], respectively. The maximum thresholds are selected to separate the interesting objects from their edges. As can be seen in Figs. 2, 3, 7, and 8, $\sigma _{max}^{PV}$ and $\sigma _{max}^{CV}$ also can be a value approximately between [1 3] and [0.5 1], respectively. It is worth mentioning that $N_{itr}^{Tot}$ is the total possible number of iterations that can be run by the hierarchical block division algorithm.

As can be seen in Fig. 10, this algorithm is run in four major steps:

- Step 1: Create two empty lists (*L*_1_ and *L*_2_) and assign the entire depth map image as one block to *L*_1_. Then, start the first iteration as explained in step 2.
- Step 2: Check if the algorithm reaches the limit of $N_{itr}^{Tot}$ or if all blocks in *L*_1_ are with *PV* or *CV* values smaller than $\sigma _{max}^{PV}$ or $\sigma _{max}^{CV}$, respectively. If yes, go to step 4. If not, go to step 3. Clearly, in the first iteration, there is only one block in *L*_1_ and its metrics are with the strong probability greater than *σ*_*max*_.
- Step 3: For every block in *L*_1_ with the metric value greater than the threshold, divide the block into four equal-sized blocks and assign them to *L*_2_. Any block with a metric value less than the threshold is assigned without change to *L*_2_. After having checked all the blocks in *L*_1_, *L*_1_ is updated with *L*_2_ and *L*_2_ is cleared. Then, return back to step 2.
- Step 4: All blocks in *L*_1_ with metric values less than *σ*_*min*_ are considered as region I. Blocks with metric values within the ROI range are considered as region II and remainders are region III.

In the hierarchical block division algorithm, a block is partitioned into smaller blocks by dividing the width and height of the block by a factor 2 in each iteration. It is worth mentioning that $N_{itr}^{Tot}$ should be defined in order to have blocks with sizes at least greater than 2 × 1 or 1 × 2 pixel block size. This is due to the fact that both metrics used in this algorithm evaluate pixel variation where there are at least two pixels to measure the variation.

**Fig. 11:**
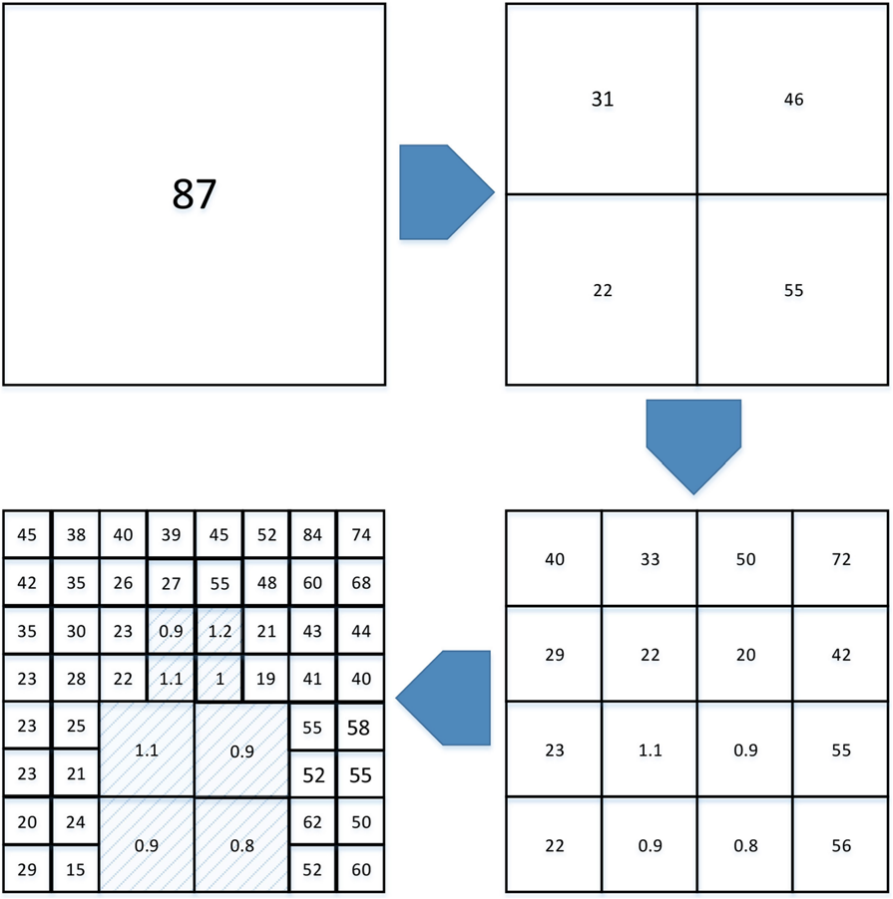
Sample hierarchical division process. In this example, it has been assumed that *σ*_*max*_ = 10, $N_{itr}^{Tot}~=~4$, and the size of the frame is 16 × 16. For simplicity, the ROI range is defined less than *σ*_*max*_. In the beginning, the entire frame is considered as one block. Since its metric value is greater than the metric value, it is divided into four blocks, and this process is continued until the number of iterations equals to $N_{itr}^{Tot}$ or the metric values of all blocks are in the ROI range

A sample hierarchical block division process is shown in Fig. 11. You may assume that the numbers inside the blocks represent typical values for the metric values used in the algorithm. For this figure, it also has been assumed that the resolution of the depth map image is 16 × 16 pixels and the smallest block is of the size 2 × 2. Clearly, $N_{itr}^{Tot}$ is 4 and *σ*_*max*_ can be assumed as 10. The highlighted blocks in the fourth iteration are showing the important region of the frame that the proposed algorithm is looking for. As can be seen in this example, there are some blocks with large metric values resulting in further partitioning, but because the number of algorithm iteration reaches $N_{itr}^{Tot}$, the algorithm stops partitioning blocks. In this example, *σ*_*min*_ was not defined, but in practice, this parameter should be used in order to separate very far background objects with very small depth values (close to zero) from interesting objects in the depth map image. Because the background region is often out of focus during the capturing of videos, this background region also needs to be excluded from ROI in the proposed algorithm. Performances of the ROI extraction algorithm for the metrics *PV* and *CV* are examined and compared in Section 3.

### MDC Polyphase SubSampling algorithm

**Fig. 12:**
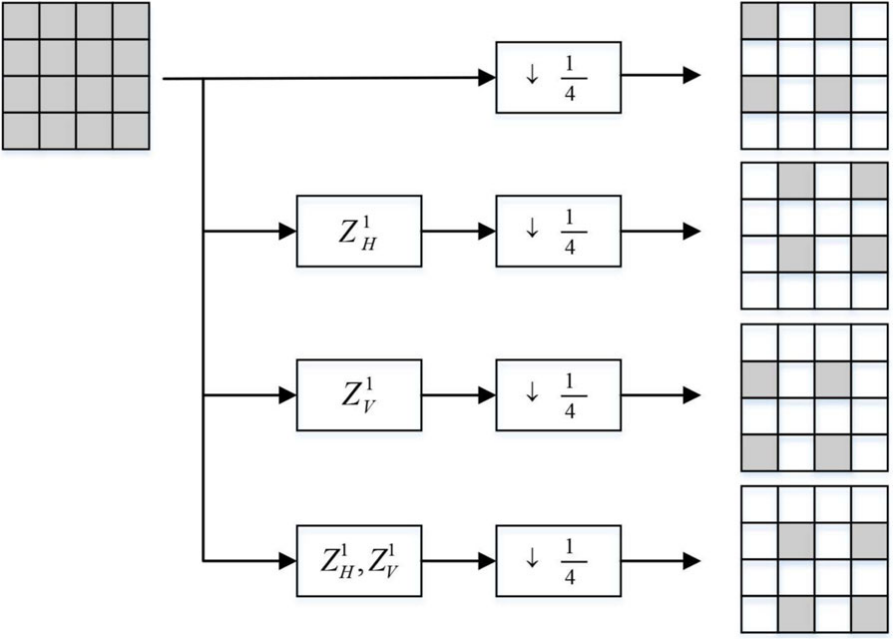
Polyphase SubSampling MDC encoder $Z_{H}^{1}$ and $Z_{V}^{1}$ are horizontal and vertical shift, respectively

To have reliable video streaming, the proposed new spatial MDC algorithm exploits the multiple description coding (MDC) strategy for 3D videos. To this end, four descriptions are created using Polyphase SubSampling (PSS). PSS-MDC is the basic low complex method that can be used in the spatial domain to have a reliable transmission in the error-prone environment. As can be seen in Fig. 12, with the PSS-MDC encoder used by the proposed method, every description includes one of 2 × 2 pixels. Since the new spatial MDC algorithm is applied on 3D stereoscopic videos, the PSS-MDC encoder needs to be applied to both color and depth map frame separately.

### Description enhancement algorithm

Although the most important advantage of the PSS-MDC encoder is its simplicity, there is a capability lack in increasing the redundancy in order to avoid errors in the strong noisy environment. To fix this, the new spatial MDC algorithm enhances the pixel resolution for areas that are less predictable and also on objects of interest that are more important to focus on.

As can be seen in Fig. 1, two different algorithms are applied to the color video and the depth map stream. For the depth map stream, the resolution of each description is enhanced according to its prediction difficulty. Since the metrics defined in this paper evaluate the variation between adjacent pixels, it can be said that pixels of the depth map frame are clustered into regions I to III according to their difficulty prediction levels. This means that region I, which includes pixels with very low variations, remains without any change. Pixel resolution in region II is enhanced to half resolution (of the original resolution) for each description in the encoder by picking any pixels of 2 × 2 pixels other than the pixel that was initially assigned to the description. Since it has been assumed that three descriptions are lost due to the unreliable communication and only one description is available in the decoder, it is of minor importance which pixel is added to the pixels in the one fourth resolution. Since region III contains pixels with large variations, it is likely that the prediction of a pixel (in case of missing) from adjacent pixels leads to error. As a result, this region’s pixel resolution has increased to a fuller pixel resolution for each description.

Since the region’s clustering algorithm is done using the depth map image rather than the color video frame, it cannot reflect the pixels’ value variations for the color video frame. Therefore, the abovementioned argument is no longer applicable. One suggestion with regard to the color video is to apply the proposed ROI detection algorithm on the color video stream in order for it to extract ROI map based on the pixel variation found in the color video frame, but the drawback is its greater complexity due to a wide variety of colors inherently part of any scene naturally. As a result, the hierarchical block division algorithm needs more time to identify different regions in the frame. Another suggestion is to use the ROI map extracted from the depth map image to then focus on region II for the enhancement of pixel resolution in the color video frame rather than on region III which is performed within the depth map stream. Since the human eye is more sensitive to objects rather than of pixels, this suggestion introduces better performance with regard to the subjective assessment. Also, it can provide improvement with regard to the objective assessment since the recording of moving objects, which are inherently part of the scene, are now more focused. Because all video coding standards use differential pulse code modulation (DPCM) and proximate pixels’ values of the objects in the color video frame, the increase of the resolution of those parts of a frame that include the ROI can be compensated by DPCM algorithm in point of compression ratio. Therefore, with regards to the color video stream, regions II and III are enhanced to full and half resolution, respectively. Region I remains with the same resolution as before (one fourth). This enhancement algorithm helps to perfectly recover the ROI in the instance of missing a description, although at the expense of increased redundancy.

## Simulation result and discussion

**Fig. 13:**
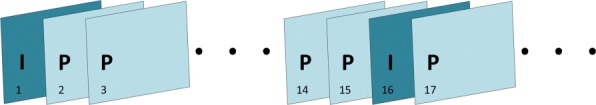
Key frame structure. A sequence of video frames, consisting of two key frames labelled as “I” at the first and 16th frame and 15 forward-predicted frames labelled as “P” in between of the key frames

For the evaluation of the proposed algorithm, this paper carried out several tests using two stereoscopic video sequences with the format of DVD-Video PAL (720×576), called video “Interview” and “Orbi.” The chroma and depth subsampling format is 4:2:2:4 (the last 4 shows the resolution of the depth map image) or in other words, the total frame resolution is 1440×576. Each video has 90 frames and the frame rate is 30 frames per second (fps). The new algorithm is implemented using H.264/AVC reference software, JM 19.0 [23]. To encode with JM software, *I* frames are repeated every 16 frames and only *P* frames are used between *I* frames, as shown in Fig. 13.

As described in the previous section, the hierarchical block division algorithm halves both width and height dimensions of a block in each iteration to make smaller blocks. It should be noted that for the mentioned video sequences, the width of the depth map frame (720=2^4^ × 3^2^ ×5) is not divisible after the fourth iteration. To be able to continue the hierarchical block division algorithm after the fourth iteration, we extend the depth width to 768 (=  2^8^ × 3) (add zeros to the left side of the depth map image). With the same argument, the height of the depth map frame is assumed to be 512 (=  2^8^×2). Therefore, the acceptable minimum size of a block at the end of the hierarchical division algorithm is 2 × 3 which is achieved after the eighth iteration (this means that the $N_{itr}^{Tot}$ equals 8 and the minimum block size after the eighth iteration in the hierarchical block division algorithm is 6 pixels). The minimum and maximum thresholds used for the tests presented in this paper are $\left [\sigma _{min}^{PV}~=~0.3,\sigma _{max}^{PV}~=~3\right ]$ and $\left [\sigma _{min}^{CV}~=~0.01,\sigma _{max}^{CV}~=~0.5\right ]$. In the remainder of this paper, we will first discuss the complexity of the proposed algorithm in general. Then, both the performance and complexity of the proposed algorithm using *PV* and *CV* will be compared. Thereafter, we will evaluate the performance of the new proposed spatial MDC algorithm for streaming in the error-prone environment. It is worth mentioning that to simulate an error-prone environment, we have assumed that the decoder receives only one description among four descriptions generated by the encoder.

### Visual performance examine

**Fig. 14:**
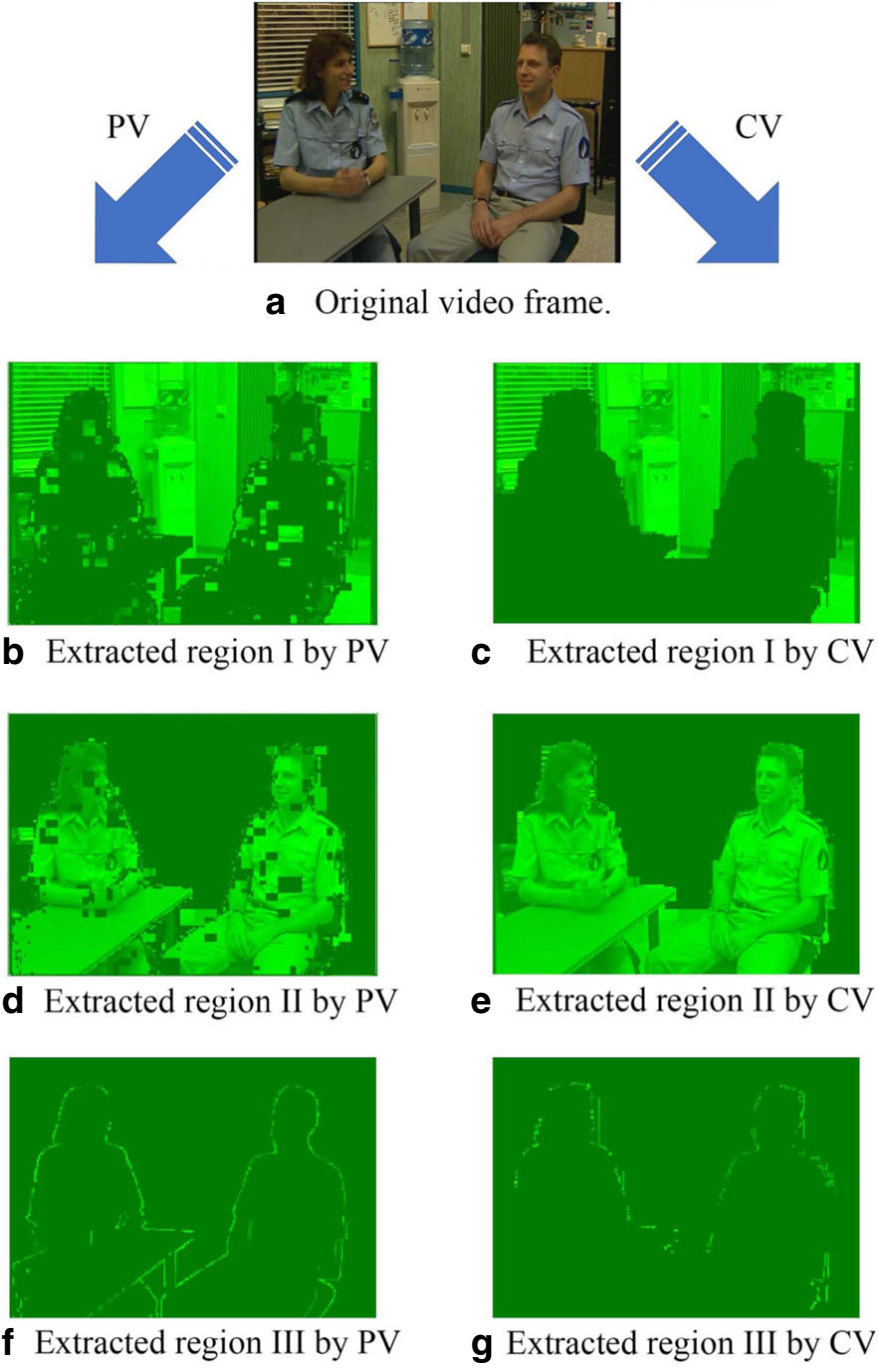
Performance comparison of *PV* and *CV*. Left figures show the identified regions I–III using *PV*, and right figures show the regions using *CV* for the first frame of video “Interview”. As can be seen for the region III, using *PV* is more accurate rather than using *CV*, while for the region III, *CV* outperforms compared to *PV*. **a** Original video frame. **b** Extracted region I by PV. **c** Extracted region I by CV. **d** Extracted region II by PV. **e** Extracted region II by CV. **f** Extracted region III by PV. **g** Extracted region III by CV

Figure 14 shows the identified regions I to III using *PV* and *CV* metrics. Clearly, the identified region II is more accurately depicted with the *CV* metric rather than with the *PV* metric. The same scenario is also applicable to the region I. As can be seen in part d of Fig. 14, there are some important pixels that have not been identified as the region II (ROI). Also, we have detected some missed pixels in region I (background) with *PV* as shown in part b of Fig. 14. Such inaccuracy in identifying different regions with *PV* can be due to the fact that pixel values of different blocks are in dissimilar ranges. Therefore, the pixel variation (*PV*) cannot be an appropriate metric to be used when extracting for regions I and II. To fix this problem as argued before, it is necessary to normalize the pixel variation metric(*PV*). Indeed, the *CV* metric is the normalized pixel variation and works like a smoothing filter. Although using normalized pixel variation metric (*CV*) provides a considerable improvement in the extraction of regions I and II, such performance is not shown when using the *CV* metric in identifying region III (which stands for the edges). As can be seen in Fig. 14, the detected edges shown in part g are not as clear as the detected edges shown in part f. The reason for that can be the smoothing effect brought about by the normalization using the *CV* metric. As the blocks that contain edges are considered as blocks with high-frequency contents, a high-frequency filter like the pixel variation measurement (*PV*) is more beneficial for identifying the edges.

### Complexity performance examine

Table 1 shows the average number of blocks in different sizes after the hierarchical division algorithm for the videos Interview and Orbi. As can be seen, there is around one block with the size of 24576 (=  128×192) using *PV* metric and around two blocks of this size using *CV* metric for both test video sequences. This means that about 5∼9*%* of the entire depth map image is excluded from being more partitioned and stopped after the second iteration in the hierarchical division algorithm. Considering the second large block size for *PV* metric in Table 1, i.e., 6144 (=  64×96), it can be said that the hierarchical division process will be stopped for more than one third of the entire depth map image in the video Interview and one fourth of the depth map image in video Orbi after the third iteration. This result shows that the hierarchical division algorithm does not give rise to a high load of calculation in this proposed algorithm. Comparing the results using *CV* metric and *PV* metric, also shows that the complexity of using *CV* metric is lower than using *PV* metric. As can be seen, the largest block size for the *CV* metric is increased to 98304 (=  256×384) and the average number of blocks with the size of 24576 is two in the video Interview and three in video Orbi for metric *CV*.

**Table 1:** Number of blocks with different sizes after hierarchical division algorithm using metrics *PV* and *CV*

	Metric *PV*		Metric *CV*	
Blocks’ size	No. of blocks	Percent	No. of blocks	Percent
Video “Interview”				
6 (2 × 3)	3401.467	5.190	2168.222	3.308
24 (4 × 6)	994.611	6.071	358.256	2.187
96 (8 × 16)	509.156	12.431	169.322	4.134
384 (16 × 24)	209.711	20.480	81.267	7.936
1536 (32 × 48)	47.722	18.641	47.078	18.390
6144 (64 × 96)	20.644	32.257	25.433	39.740
24576 (128 × 192)	0.789	4.931	3.622	22.639
98304 (256 × 384)	0.000	0.000	0.067	1.667
Video “Orbi”				
6 (2 × 3)	5253.556	8.016	2264.044	3.455
24 (4 × 6)	1056.300	6.447	378.167	2.308
96 (8 × 16)	504.867	12.326	160.000	3.906
384 (16 × 24)	177.322	17.317	72.500	7.080
1536 (32 × 48)	73.711	28.793	51.611	20.161
6144 (64 × 96)	11.167	17.448	12.956	20.243
24576 (128 × 192)	1.544	9.653	1.611	10.069
98304 (256 × 384)	0.000	0.000	1.311	32.778

Table 2 shows the average number of blocks for different metric values of *PV* and *CV*. As can be seen, about 55% of the depth map image for video Interview and 40% of the depth map image for the video Orbi have *PV* values less than 1. On the other hand, for the video Interview, more than one half and for video Orbi more than one third of the depth map image have very close depth values. This is the reason why the decimation of the depth map image does not affect its quality when it is reconstructed in the decoder (as discussed earlier, Karim et al. showed through its simulation results that the decimation of the depth map image does not cause any considerable degradation in the decoder [22]). Table 2 also shows that about 95% of the depth map image for both test video sequences have *PV* values less than 3. The fact that about 95% of the depth map image have similar depth values result in no longer needing to send the depth map image with its original resolution, justifying why the nonidentical decimation is more advantageous than the identical decimation suggested by Karim et al. in [22]. On the other hand, only about 5% of the depth map image needs to be encoded with the original resolution. The 95% remainder can be decimated to save bandwidth or storage.

**Table 2:** Number of blocks with different metric values after hierarchical division algorithm

		Blocks’ size								Percent of blocks with
		6	24	96	384	1536	6144	24576	98304	metric value in a
		(2 × 3)	(4 × 6)	(8 × 16)	(16 × 24)	(32 × 48)	(64 × 96)	(128 × 192)	(256 × 384)	specific range(%)
Video “Interview”										
PV	≤ 1	662.78	371.67	172.54	75.80	22.28	17.82	0.68	0.00	55.68
	1∼3	1008.44	618.18	336.37	133.91	25.44	2.82	0.11	0.00	41.64
	3∼10	831.50	4.77	0.24	0.00	0.00	0.00	0.00	0.00	1.30
	≥ 10	898.74	0.00	0.00	0.00	0.00	0.00	0.00	0.00	1.37
CV	≤ 0.1	646.10	276.93	150.99	67.53	37.82	18.57	0.00	0.00	56.74
	0.1∼0.2	32.79	11.21	4.24	2.59	2.59	1.28	1.93	0.00	15.57
	0.2∼0.3	45.37	16.27	5.67	3.80	3.92	2.40	0.00	0.00	5.96
	0.3∼0.4	105.10	24.84	4.11	3.00	2.19	2.34	0.00	0.00	5.22
	0.4∼0.5	52.64	29.00	4.31	4.34	0.56	0.84	1.69	0.07	14.55
	≥ 0.5	1286.22	0.00	0.00	0.00	0.00	0.00	0.00	0.00	1.96
Video “Orbi”										
PV	≤ 1	542.72	295.40	172.56	69.13	34.42	4.69	0.80	0.00	39.37
	1∼3	1680.86	752.81	331.84	108.19	39.29	6.48	0.74	0.00	55.95
	3∼10	2276.38	8.09	0.47	0.00	0.00	0.00	0.00	0.00	3.53
	≥ 10	753.60	0.00	0.00	0.00	0.00	0.00	0.00	0.00	1.15
CV	≤ 0.1	614.22	244.82	118.68	49.28	35.30	6.58	0.81	0.00	39.28
	0.1∼0.2	59.43	28.10	11.99	8.81	5.51	1.64	0.10	0.00	6.76
	0.2∼0.3	79.78	28.24	9.36	5.84	4.41	2.56	0.48	0.40	19.80
	0.3∼0.4	134.88	35.41	10.13	4.74	4.08	1.38	0.22	0.61	21.54
	0.4∼0.5	90.23	41.59	9.84	3.82	2.31	0.80	0.00	0.30	10.66
	≥ 0.5	1285.50	0.00	0.00	0.00	0.00	0.00	0.00	0.00	1.96

Table 3 compares the statistics of the blocks generated by the hierarchical division algorithm using two metrics *PV* and *CV*. As shown in the table, the average block size for videos Interview and Orbi after hierarchical division algorithm is greater, and the average number of blocks is considerably less when the *CV* metric is used. This means fewer operations are required to identify the final blocks using the *CV* metric. It also should be considered that the results obtained by the *CV* metric have a greater performance when compared to the results gained by the *PV* metric (see Fig. 14). Therefore, better performance and less complexity can be achieved by using the new *CV* metric.

**Table 3:** Blocks statistics after hierarchical division algorithm

	Video	Interview	Orbi
Metric *PV*	Average number of blocks per frame	5184.1	7078.47
	Average block size	4.08 × 6.12	3.51 × 5.27
	Average of *PV* values	6.86	4.3
Metric *CV*	Average number of blocks per frame	2853.27	2942.2
	Average block size	4.22 × 6.33	3.91 × 5.86
	Average of *CV* values	0.548	0.539

### Robustness performance examine

**Fig. 15:**
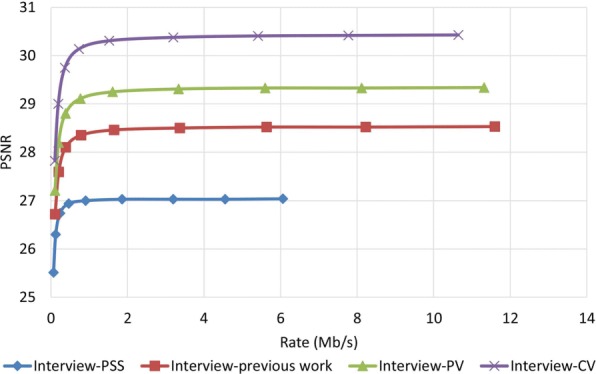
PSNR assessment of the color image for video Interview

**Fig. 16:**
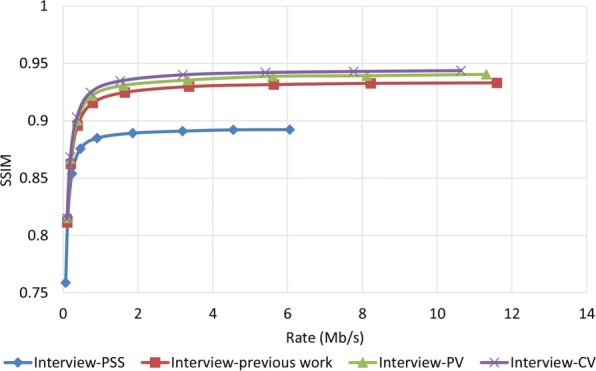
SSIM assessment of the color image for video Interview

**Fig. 17:**
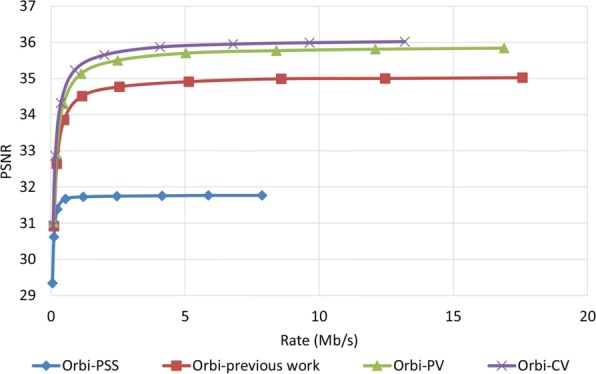
PSNR assessment of the color image for video Orbi

**Fig. 18:**
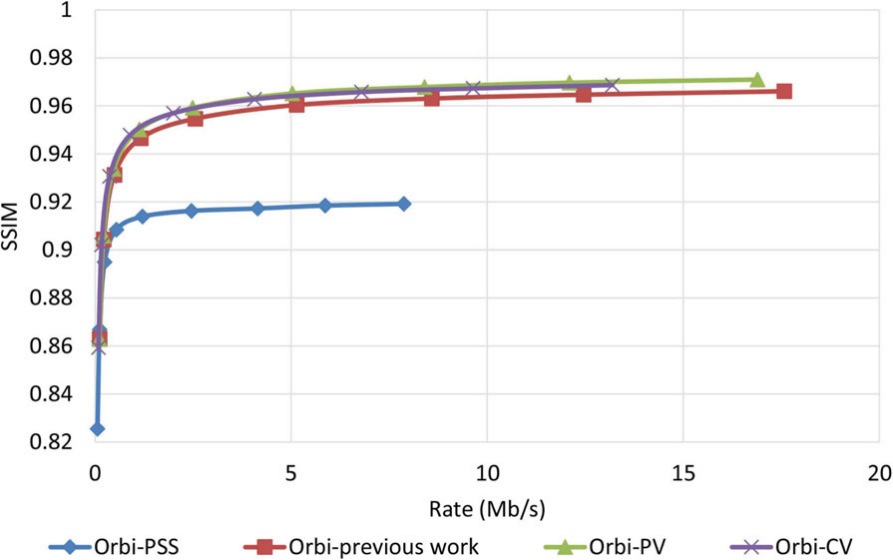
SSIM assessment of the color image for video Orbi

To examine how robust the proposed method is against error, we assumed that the decoder has access to only one description and all other three descriptions have been corrupted. In order to reconstruct the video, the decoder estimates the missed pixel from the nearest available pixel. Figures 15 and 16 compare PSNR and SSIM measurements of the reconstructed color video for video Interview using the basic Polyphase SubSampling MDC method (PSS-MDC), our previous MDC method presented in [24], and the new proposed spatial MDC algorithm with the help of *PV* and *CV* metrics. Figures 17 and 18 also show the PSNR and SSIM assessments for the video Orbi. As can be seen in Fig. 15, in the recreated video Interview, about 1 dB improvement for the *PV* metric and 2 dB improvement for the *CV* metric can be achieved by the new proposed spatial MDC algorithm when compared to our previous work presented in [24]. Regarding video Orbi (see Fig. 17), although a considerable improvement cannot be seen compared to our previous work, more than 2 dB improvement has been achieved by the new proposed spatial MDC algorithm in comparison with the PSS-MDC method. It should be noted that the complexity of implementing the proposed method is approximately halved according to Table 3. In this table, the hierarchical block division algorithm for the *PV* metric is the same as the algorithm presented in [24]. Since the average number of blocks is about 1 s and the average size of blocks is greater for the *CV* metric compared to the *PV* metric, we have concluded that the new proposed spatial MDC algorithm using the *CV* metric is more efficient. Regarding the SSIM assessment, the proposed algorithm provides about 0.3 improvement for both test videos in high rate streaming compared to the PSS-MDC method. It should be mentioned that since the human eye is more sensitive to objects rather than pixels, a subjective assessment can better emphasize the improved performance brought forward by the proposed algorithm compared to the previous methods.

**Fig. 19:**
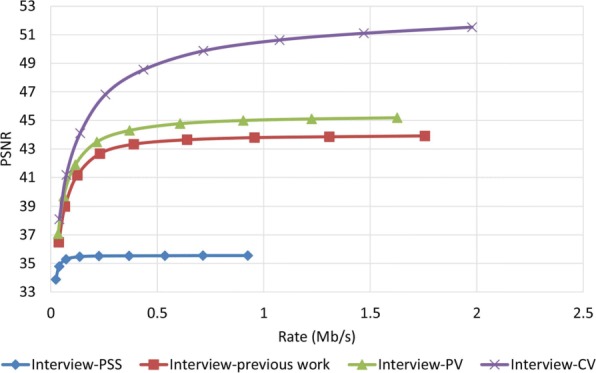
PSNR assessment of the depth map image for video Interview

**Fig. 20:**
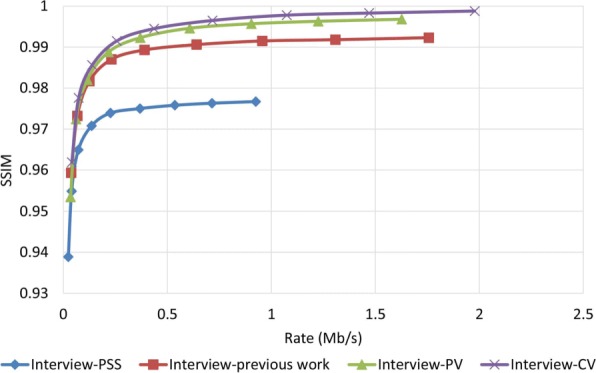
SSIM assessment of the depth map image for video Interview

**Fig. 21:**
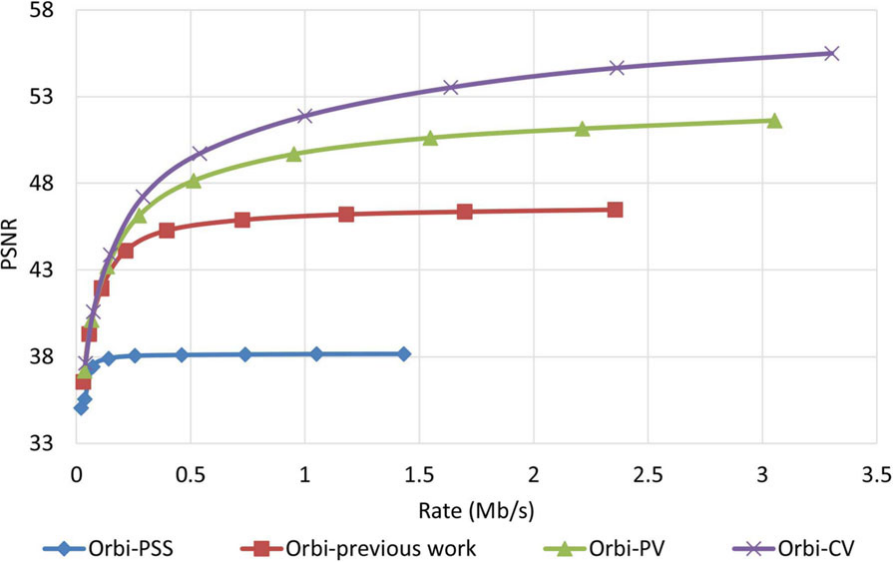
PSNR assessment of the depth map image for video Orbi

**Fig. 22:**
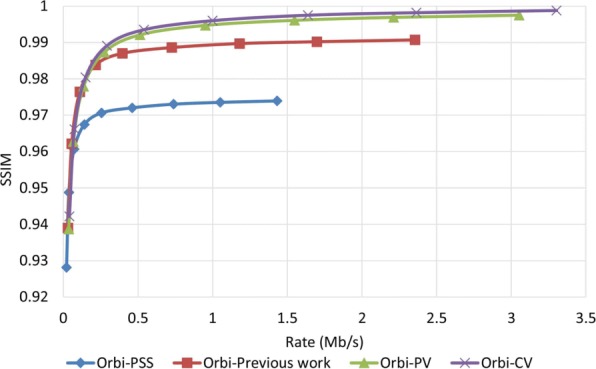
SSIM assessment of the depth map image for video Orbi

When it comes to the evaluation of the proposed algorithm for the reconstructed depth map image, it shows a better performance. As shown in Figs. 19 and 20 for the video Interview and in Figs. 21 and 22 for the video Orbi, the improvement of the proposed algorithm is considerably evident. This can be due to the fact that metrics *PV* and *CV* are calculated based on the depth map image, and therefore, blocks with larger values of metrics *PV* and *CV* can be considered as the least predictable blocks in the depth map image. Therefore, focusing on these pixels in each description results in a more accurate reconstruction in the decoder. In view of the PSNR assessment, about 8 dB for video Interview and more than 10 dB for video Orbi improvement have been achieved by the proposed algorithm. Such high performance of the proposed algorithm in view of the SSIM assessment is also more evident compared with the color video assessment. With regards to the SSIM assessment, the proposed algorithm outperforms by more than 0.02 compared to the PSS-MDC method.

**Fig. 23:**
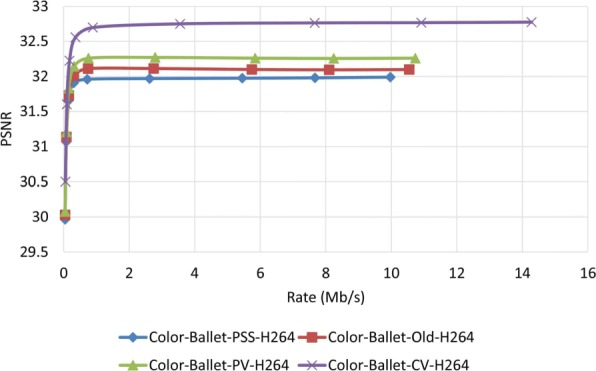
PSNR assessment of video Ballet using H.264 encoder

**Fig. 24:**
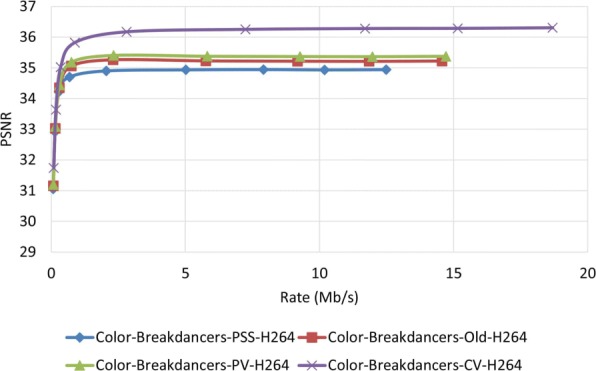
PSNR assessment of video Breakdancers using H.264 encoder

**Fig. 25:**
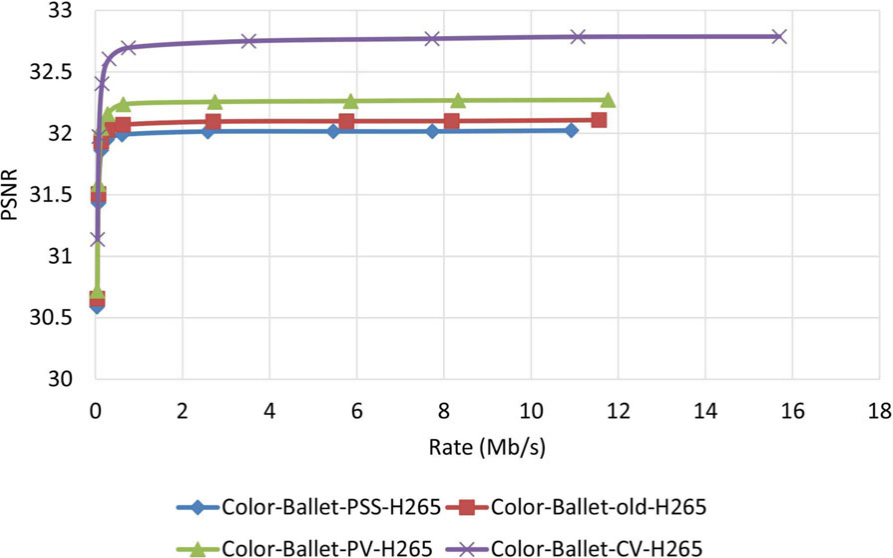
PSNR assessment of video Ballet using H.265 encoder

**Fig. 26:**
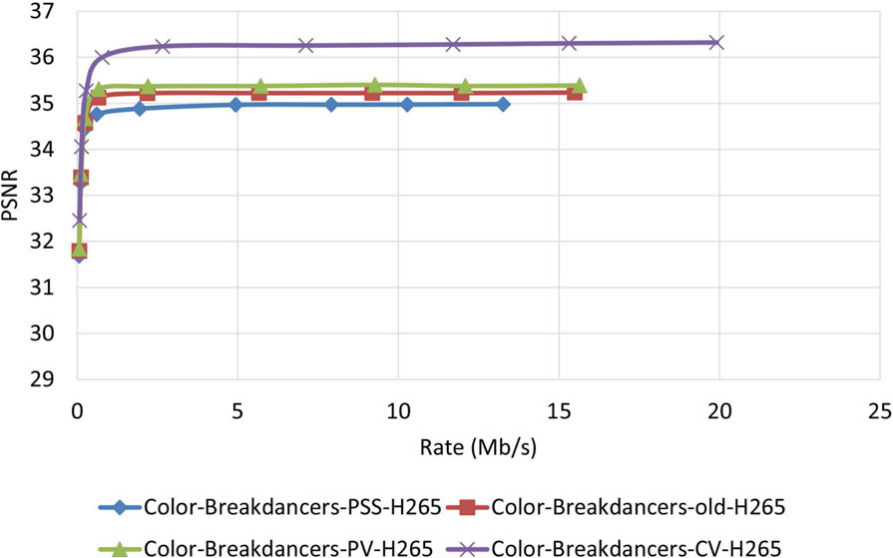
PSNR assessment of video Breakdancers using H.265 encoder

Figures 23 and 24 show the PSNR assessment for two different test video sequences, called “Ballet” and “Breakdancers,” generated by the interactive visual media group at Microsoft research [25]. Unlike the previous test video sequences, the new test video sequences include objects with very fast movement. As can be seen in these figures, like previous experiments, the proposed MDC method provide improved performance. Figures 25 and 26 demonstrate the PSNR assessment of these two test video sequences using the most recent video encoder, i.e., H.265/HEVC. To implement H.265/HEVC encoder, we used H.265 reference software, HM 6.0 [26].

## Conclusions

Multimedia streaming is affected by packet failure in the network due to packet loss, packet corruption, and large packet delay. An appropriate solution against packet failure in the error-prone environment can be multiple description coding (MDC). With MDC, one video description is partitioned into several separately decodable descriptions. In the instance of missing a description during transmission, the decoder is capable to estimate the lost description from other error-free description(s). To improve the basic spatial partitioning and to be applicable to 3D videos, a nonidentical decimation algorithm for the stereoscopic videos has been provided in this paper. Our algorithm works based on existing objects in the scene and assigns more bandwidth to the region of interest. Since human eyes are more sensitive to the objects rather than pixels, the proposed algorithm can provide an improved performance compared to the PSS-MDC method in view of subjective assessment. However, the objective assessment results confirm the improved performance achieved by the proposed spatial MDC algorithm. With regard to the depth map image, the proposed algorithm enhances the current basic decimation to a nonidentical decimation. As shown earlier, most parts of the depth map have similar depth values, and therefore, decimation in those parts can save bandwidth or storage without considerable quality degradation. However, for the parts of the frame with high pixels’ value variation, it is recommended to keep the original resolution. Therefore, with the new algorithm, those parts of the depth map image that have large variations are encoded with the original resolution.

## Abbreviations

3D: Three dimensional
ARQ: Automatic Repeat reQuest
CDF: Cumulative Density Function
CV: Coefficient of variation
DCT: Discrete Cosine Transform
ERC: Error-Resilient Coding
FEC: Forward Error Correction
HD: High definition
HEVC: High-Efficiency Video Coding
LC: Layered coding
LOT: Lapped Orthogonal Transformation
MDC: Multiple description coding
PDF: Probability Density Function
PSS: Polyphase SubSampling
PSNR: Peak signal-to-noise ratio
PV: Pixels variance
ROI: Region of interest
RVLC: Reversible Variable Length Coding
SSIM: Structural SIMilarity
